# Perceiving the affordance of interceptability for another

**DOI:** 10.3389/fpsyg.2025.1566278

**Published:** 2025-06-03

**Authors:** Samruddhi Damle, Reinoud J. Bootsma, Frank T. J. M. Zaal

**Affiliations:** ^1^Department of Human Movement Sciences, University Medical Center Groningen, University of Groningen, Groningen, Netherlands; ^2^Institut des Sciences du Mouvement, Aix-Marseille Université, CNRS, Marseille, France

**Keywords:** perception-action, interception, interceptability, affordance, affordance for another

## Abstract

Previous research has established that people can make accurate perceptual judgments regarding the affordance of interceptability for oneself. The present study aimed to explore whether people are also capable of perceiving interceptability for another person. Using a manual lateral interception paradigm, we examined whether a group of observers could make perceptual judgments about the affordance of interceptability for a particular individual (the “actor”). We additionally explored the effects of prior training and of partial visual occlusion on the perception of interceptability for the actor. Three groups of 12 observers each viewed the ball-and-paddle kinematics of the actor performing the interception task. Two groups received full vision, whereas one group received partially occluded vision of the screen. Two groups also received prior training on the interception task, whereas one group did not. All observers were required to make verbal judgments (“no”-calls) when they perceived a ball to be uninterceptable for the actor. The frequency and timings of the judgments of the observers turned out to be similar to those of the actor. Analogous task variables characterized the perceptual performance for the observers and actor alike, suggesting that observers were indeed capable of perceiving affordances for the actor. Lastly, we found that neither prior training, nor visual occlusion, had any significant impact on the observers' judgments. We concluded that individuals are capable of perceiving action possibilities for another person, in a comparable way as they would for themselves.

## 1 Introduction

Every moment of the day, our actions are shaped by affordances—here operationally defined as action possibilities for individuals (Gibson, 1979/1986; Baggs and Raja, 2024; Bruineberg et al., 2024)—for ourselves and for others. Often, involving others is part and parcel of how we act (e.g., Benerink et al., 2016; Richardson et al., 2007). The ability to perceive one's own action boundaries and limits is well-documented across various tasks, such as sitting, reaching, jumping, and climbing (e.g., Wagman, 2019; Mark, 2007; Warren, 1984; Seifert et al., 2018; Mark, 1987; Thomas et al., 2018). These studies have typically focused on affordances under static conditions. That is to say, at the time scale of the experiments in these studies, action boundaries remained stable (e.g., in judging climbability, stair risers did not change height during trials). However, recent research has extended this understanding to affordances under dynamic conditions, when opportunities for action may vanish over the course of a trial (Damle et al., 2024; Fajen and Matthis, 2011; Postma et al., 2017, 2018, 2022; Oudejans et al., 1996).

The present study builds on previous work (Damle et al., 2024) in which we studied the affordance of interceptability for oneself. That study adopted a virtual lateral-interception task (cf. Ledouit et al., 2013, 2024) in which balls that moved down across a large computer screen could be intercepted with a virtual paddle. Participants used a physical slider, placed in front of them, to control an on-screen paddle that could move along an invisible horizontal interception axis near the bottom of the screen. Most of the balls were interceptable but a number of balls was designed to be uninterceptable. The study showed that the interceptability of a ball was related to the distance that had to be traveled with the paddle to the interception location, the ball's flight time, and the angle under which the ball approached the interception axis. Furthermore, when participants were allowed to abandon an interception attempt after indicating verbally that the interception would not be possible, they turned out to be quite proficient in perceiving interceptability.

People can perceive interceptability for themselves (Damle et al., 2024; Fajen et al., 2011; Postma et al., 2018). Are they also able to perceive interceptability for somebody else? In our social world, we need not only to be able to perceive our own action capabilities and boundaries, but also those of others. Previous work has described the ability of individuals to perceive affordances for others in a variety of situations. Such results range from perceiving affordances of maximum sitting height for another person (Stoffregen et al., 1999; Mark, 2007; Weast et al., 2011) to gap-crossing and climbing stairs (Mark, 2007), and jumping-to-reach (Ramenzoni V. C. et al., 2008; Ramenzoni V. et al., 2008; Ramenzoni et al., 2010). Mark (2007) highlighted that people can perceive the critical action boundaries for other individuals with as much accuracy as they would for themselves when perceiving certain affordances.

In sum, a number of studies have demonstrated that people, indeed, are able to perceive affordances for another person. Is this also the case for the affordance of interceptability, an opportunity for action that may vanish as a function of both organismic factors (e.g., the time that someone needs to start moving) and environmental factors (e.g., the time remaining for a ball to arrive at the potential interception location)? Despite the widespread array of findings on affordance perception for others, there remains a lack of focus on perceiving affordances for others under dynamic environmental conditions. Yet, we often encounter situations which tend to be more dynamic in nature, such as in sports settings or in crossing a street. For instance, in a double's tennis match, knowing whether a ball can be intercepted by one's teammate is as important as knowing whether one can intercept the ball oneself (Benerink et al., 2016, 2018; Van Opstal et al., 2018). Similarly, it is crucial for parents to be able to perceive their children's action capabilities and limits in high-risk environments, as demonstrated by Cordovil et al. (2012) in swimming scenarios.

To address the perception of interceptability for another person, in the present contribution we presented to the participants (hereafter referred to as the observers) played-back ball and paddle kinematics recorded from interceptive actions performed by one of the participants of the Damle et al. (2024) study (hereafter referred to as the “actor”). Analogous to the instructions given to the original actor (call “no” as soon as the ball looks uninterceptable), the instructions to the observers were to signal (call “no”) when they perceived the ball to be uninterceptable for the original actor, that is, the person who controlled the paddle in the displays that we presented. Using Generalized Linear Mixed Effects Regression, Damle et al. (2024) analyzed which task variables (co)determined the (un)interceptability of the balls as well as how accurate the “no”-calls of the participants were. The participant that we selected as actor in the present study called her “no” for uninterceptability on a large number of trials while the analyses indicated that her accuracy was on par. In the present study, as a first step to understanding the perception of affordances for another, we examined whether the pattern of “no”-calls of the observers was structured in the same way as that of the actor, in terms of their dependency on the task variables.

In addition to examining the primary question of affordance perception for another, we explored two related aspects: the role of prior training on the task and that of partial visual occlusion of the display. Evidence suggests that prior experience, practice and/or training tend to improve one's own affordance perception (Franchak et al., 2010; Yasuda et al., 2014; Stoffregen et al., 2009; Seifert et al., 2018). However, whether this also extends to perception of affordances for another is unclear. Several studies suggest that this might be the case. For instance, compared to non-basketball players, expert basketball players are better at indicating the critical height that another person can reach when allowed to jump, an action relevant in basketball (Weast et al., 2011; see also Casile and Giese, 2006, for a similar demonstration in discriminating rhythmical biological motion). To address this, we examined whether prior personal practice (hereafter referred to as training) on the task can impact perceiving affordances for another person. Secondly, it has not been established whether knowing the outcome of the trial (knowledge of results) affects the perception of affordances. Typically, in studies on the perception of static affordances, judgments and actions are collected in separate sessions (Wagman et al., 2019; Bingham et al., 1989). In studies in which judgements are being made during actions, such as in the Damle et al. (2024) study, this is usually not possible (see also Fajen et al., 2011; Postma et al., 2018). However, an experiment with played-back prerecorded interceptive action kinematics does allow obscuring the outcome of the action. As an exploratory inquiry, we examined the effects of late-visual occlusion on affordance perception for another person. We partially obscured the visual display toward the end of the trial, thus preventing observers from seeing the trial outcome of the actor's behavior without providing any additional feedback. These two manipulations (effects of prior training and late occlusion) were chosen with the intention of exploring which factors might influence the affordance perception for another person.

## 2 Materials and method

### 2.1 Participants

We recruited 36 right-handed students (23 females, 13 males; the “observers”) from the University Medical Center Groningen, with an average age of 21 ± 2.4 years (age range 18–26 years). All provided written consent prior to participating in the study. The inclusion criteria for participants were to have normal or corrected-to-normal vision and no reported or apparent physical injuries or disabilities. Informed consent was also provided by one participant from the study by Damle et al. (2024) for using their behavioral data (movement kinematics and verbal judgments collected on the lateral manual interception task) for judgments of interceptability by others, as addressed in the present study. No other information about this person (the “actor”) was used, thus ensuring that the actor could not be identified by the participants of the present study.

The experiment was approved by the Ethics Board of the UMCG (University Medical Center Groningen, University of Groningen, Netherlands) and conducted in accordance with the Declaration of Helsinki.

### 2.2 Experimental set-up

**Figure 1:**
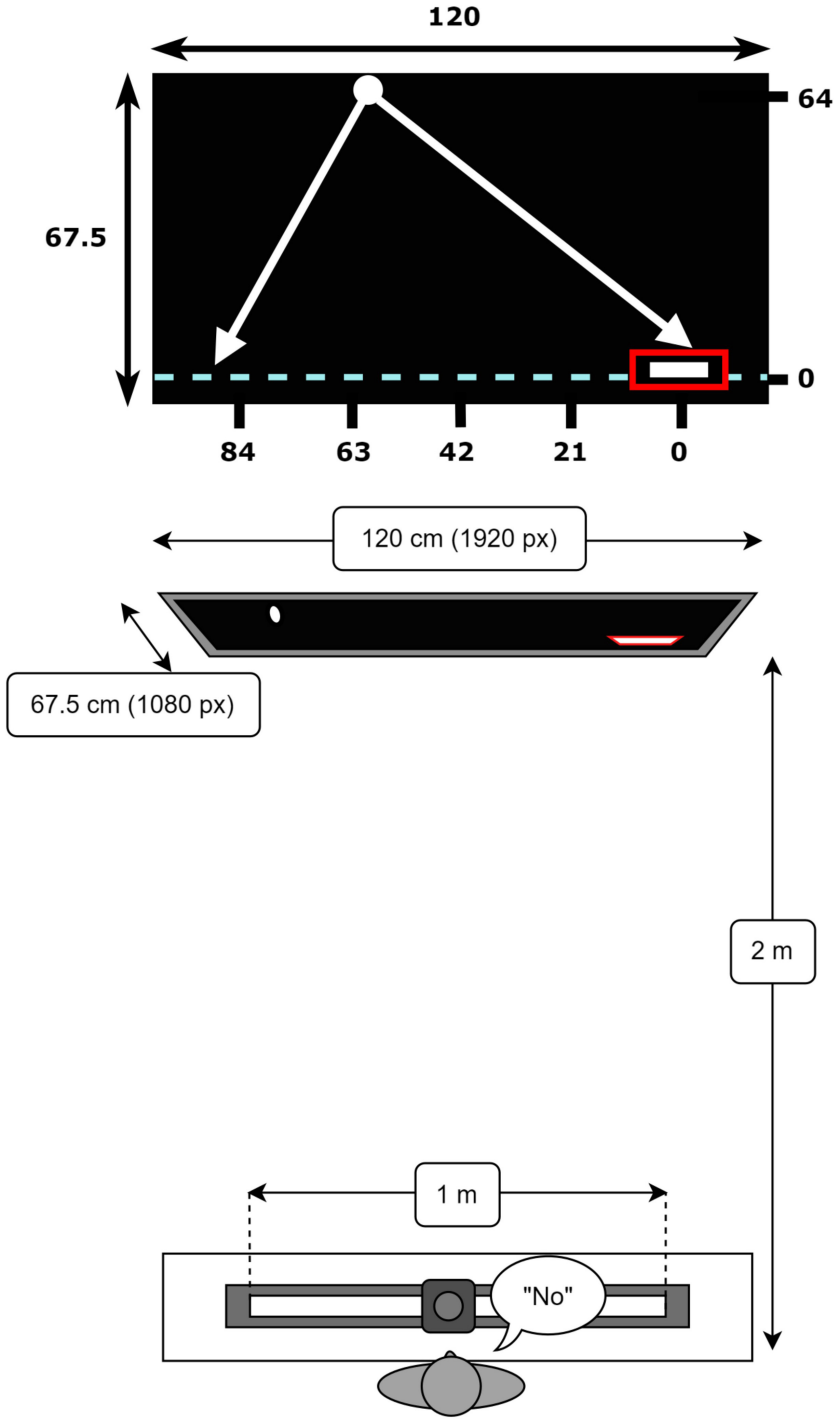
Visual representation of the screen and task dimensions in centimeters (not to scale). The fixed starting position is indicated by the red rectangle at the bottom right position on the interception axis (0,0). The five ball arrival positions are displayed on the interception axis. The axis is initialized to 0 on the right-side and in increasing order to the left so as to align with increasing distance from start. Schematic top view of the slider and TV screen.

The present study on perceiving affordances for another employed the lateral manual interception paradigm used by Damle et al. (2024). The observers sat at a desk facing a TV screen (Samsung 55” QLED QN95A, dimensions 120 × 67.5 cm, resolution of 1920 × 1080 pixels) at a distance of 2 m (Figure 1). The experiment was designed and administered using PsychoPy^®^, an open access python-based software for designing and conducting experiments (Peirce et al., 2019). We used a HP computer (Windows 11) to run the experiment. Observers' verbal judgments (“no”-calls) were recorded using a Movo M1 USB Lavalier microphone. The experimental data was stored on a secured research drive within the environment of the UMCG network.

### 2.3 Design and procedure

Each observer was randomly assigned to one of three groups, under the constraint that each group consisted of 12 participants. All three groups performed a *judging* session in which they provided judgements of interceptability for the actor. The groups differed in (i) whether they had experienced the task themselves in a *training* session before doing the *judging* session and (ii) whether in the *judging* session they saw the full or truncated (i.e., late-occluded) display of the ball and (actor's) paddle movements. Group T+Jf (Training + Judgment with full display) first performed the *training* session and subsequently performed the *judging* session under full visibility conditions. Group Jf (Judgment with full display) did not perform the *training* session but only the *judging* session under full visibility conditions. Group T+Jo (Training + Judgement with late-occluded display) first performed the *training* session and subsequently performed the *judging* session under late occlusion visibility conditions.

#### 2.3.1 Observers' judging session

The observers were required to make verbal judgments about the interceptability of virtual balls for the actor. The observers were shown real-time animations of the actor's (successful and unsuccessful) interceptive actions as recorded in our previous study (Damle et al., 2024). For these interceptive actions, the actor controlled an on-screen paddle (4.5 cm wide, 0.8-cm high white rectangle) that could move laterally along the interception axis at the bottom of the screen. A virtual ball (2-cm diameter white circle) moved from top to bottom across the black screen. The actor's task was to try to make paddle-ball contact such that the ball would bounce back up. On each trial, the actor was confronted with a ball moving at constant speed along a rectilinear trajectory downwards across the screen toward the interception axis. Combining five potential Ball Departure Positions (BDP at Y = 64 cm, X = 84, 63, 42, 21, or 0 cm) with five potential Ball Arrival Position (BAP at Y = 0 cm, X = 84, 63, 42, 21, or 0 cm, see Figure 1) gave rise to 25 different ball trajectories. To cover an entire range of BDPs and BAPs, on each trial, a random offset between −10.5 and +10.5 cm (half the distance between neighboring BDPs and BAPs) was applied to shift the entire trajectory to the left or to the right. Via adjustments in ball speed, the time it took the ball to move from the trial's BDP to the trial's BAP (i.e., Ball Flight Time: BFT) could be 1.2, 0.8, or 0.6 s. Finally, ball trajectories were extrapolated upward, such that the ball would start outside of the physical dimensions of the screen and would appear to move in from above. In each session the actor performed three blocks of trials with each block formed by fully crossing the 25 different ball trajectory conditions with the different ball flight times used in that session.

All participants in the Damle et al. (2024) study performed three sessions: a training session (with BFTs of 1.6 and 1.2 s), an action session (with BFTs of 1.2, 0.8 and 0. 6 s), and a judging session (also with BFTs of 1.2, 0.8 and 0.6 s). In the action session, the participants were instructed to attempt to intercept every ball that was presented, whereas in the judging session the participants were asked to attempt to intercept the balls but to call “no” when they perceived the ball to be uninterceptable. After calling “no” they were free to abandon their interception attempt. The animations presented to the observers in the present study were from the actor's judging session, and just as the actor, the observers were asked to call “no” as soon as they perceived a ball to be uninterceptable for the actor. The specific actor (S12, whose results are available in the rightmost panel on the fourth row in Figures 3 and 7 in Damle et al., 2024) was selected because (i) she had given consent for using the collected kinematic data in follow-up studies, (ii) her number of “no”-calls was relatively high, and (iii) the accuracy of her “no”-calls was on par. For the present study, we only used the ball trajectories with the two longer ball flight times (1.2 and 0.8 s) because the high ball speeds at the shortest (0.6 s) ball flight time turned out be quite difficult both in terms of intercepting the balls and making accurate judgments.

In the *judging* session of the present experiment, observers were shown played-back recordings of interceptive action kinematics (i.e., ball and paddle motions) for all 150 trials performed by the actor in her *judging* session under ball flight times of 1.2 and 0.8 s (25 trajectories x 2 ball flight times x 3 blocks). On each trial the paddle was initially positioned at the starting position X = 0 cm, located on the right side of the screen (see Figure 1). In watching the displays, observers who had received the training (groups T+Jf and T+Jo) could refer back to their own activity of intercepting the virtual balls, while observers who had not received training (group Jf) could not. In order to make sure that all observers understood the displays presented as well as their task, we adopted the following procedure. Before beginning the *judging* session, individual observers were informed about the lateral manual interception task performed by the actor. They were told that on each trial the actor's task had been to attempt to intercept a circular ball moving downward across the screen in front of them and that over different trials balls could follow a variety of rectilinear trajectories. We explained that, in attempting to intercept a ball, the actor controlled the position of an on-screen rectangular paddle by manually moving the knob over the slider device placed clearly visible on the table in front of them (present for all groups but not used during the *judging* session). They were informed that, over a set of trials, the actor's task had been to intercept as many balls as possible, with the set including both interceptable and uninterceptable balls. They were told that the actor had been instructed to call “no” as soon as she felt that an ongoing interception attempt would not be successful. Finally, observers were instructed that, on each trial presented to them on the screen, they were to watch the actor's unfolding interception attempt and to call “no” as soon as they perceived the ball to be uninterceptable for the actor. Thus, rather than providing us with a categorical yes/no judgment, when deemed appropriate, the observers simply indicated that they felt that interception would not be possible (anymore).

Observers with the full display (Groups Jf and T+Jf) and with the late-occluded display (Group T+Jo) were administered the same set of trials. The difference between the groups was that for the Group T+Jo the replay of trial kinematics was occluded after the ball had covered 75% of its vertical trajectory. That is, the ball and (actor's) paddle were no longer visible to the observers in the final part of each trial. In turn this meant that the final trial outcome (hit or miss) was unavailable to this group.

#### 2.3.2 Observers' training session

Before performing the *judging* task, participants from two of the three groups (i.e., Group T+Jf and Group T+Jo) first received training on the interception task (*training* session). In this session, participants actively attempted to intercept as many balls as possible themselves. The design of the *training* session was identical to the one administered to the actor in the previous Damle et al. (2024) study, using the same system of defining ball trajectories as detailed above. As had been done for the actor, the *training* session used ball flight times of 1.6 s and 1.2 s, such that all balls would be interceptable.

In the *training* session, participants thus performed three blocks of 50 trials, for a total of 150 trials. In the *training* session, participants used their right hand to move a slider that controlled the on-screen paddle. The slider-paddle system was calibrated such that extreme end positions on screen could be reached by the paddle without physically moving the slider to the extremities, that is, 90% of the slider range corresponded to 100% of the on-screen paddle range.

Each trial in the *training* session commenced with participants moving their paddle to a fixed starting position on the interception axis, depicted by a red rectangle (5.5 cm wide, 1 cm high), on the right side of the interception axis (X = 0 cm, see Figure 1). The trial began after the paddle had stayed inside the rectangle for 1 s, indicated by the ball moving down the screen. If the ball was intercepted by the paddle by bouncing it back upwards, the trial was classified as a success (marked by the paddle turning green). If the trial was unsuccessful, the ball would move downward beyond the interception axis and was classified as a miss (marked by the paddle turning red).

### 2.4 Data acquisition and analysis

The verbal judgments made by the observers in the *judging* session were recorded using a microphone. Each trial was saved as a separate wav file. An audio analysis software (Audacity^®^, version 3.4.2) was used to obtain the timestamps of the verbal calls. All the trials were synchronized to the moment the ball began to move (initialized to t = 0 s) and the timestamps of acquired “no”-calls were defined relative to this moment.

### 2.5 Statistical analysis

To analyze the actor's judgment data, we used multiple logistic regression on the presence of the actor's verbal judgments of uninterceptability (i.e., “no” calls) whereas for analyzing the observers' judgment data, we used mixed-effects regression models. The latter models account for the nested dependencies which often occur in repeated-measures designs, like in the case of the observers' data in the present study. More specifically, we used generalized linear mixed-effects regression (GLMER) for the presence of verbal judgments (i.e., “no”-calls) from the observers and linear mixed-effects regression (LMER) for the timing of the observers' verbal judgments under the different ball motion conditions.

**Figure 2:**
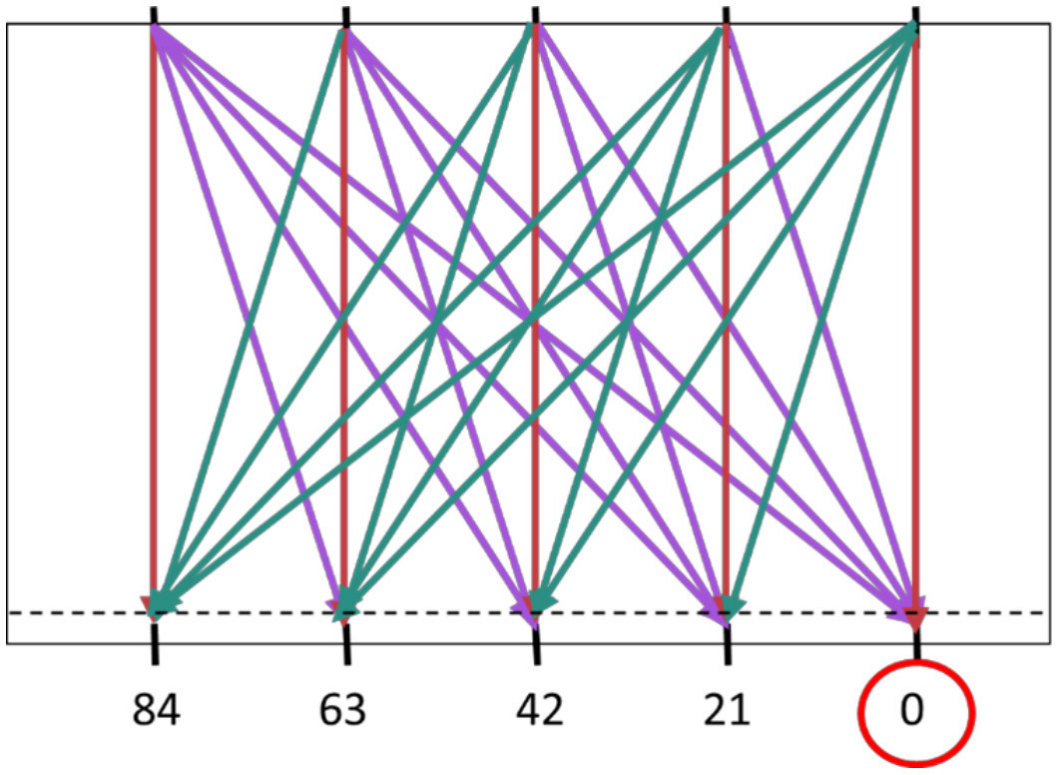
Angle of approach (AoA) defined by the angle the approaching ball makes with the vertical. The absolute angles of approach (Z-scores in brackets) were as follows: 0° (0), 17.28° (0.59), 31.89° (1.09), 43.02° (1.47), and 51.21° (1.76). Balls moving from the top right to the bottom left are classified with a positive AoA (cyan), whereas those moving from top left to bottom right have a negative AoA (purple). Balls moving orthogonally with respect to the interception axis have a zero AoA (red). Note that, on each trial, the trajectory was shifted laterally over a randomly chosen distance between −10.5 and +10.5 cm (half the distance between neighboring BDPs and BAPs), so balls could arrive on the interception axis anywhere between X = −10.5 and X = +94.5 cm.

For the GLMER model, discrete variables of ball arrival position (BAP) and ball departure position (BDP) were transformed into continuous variables of distance from start (D) and the angle of approach (AoA). These continuous variables were converted to z-scores for the purpose of the analyses. Distance from start (D) was calculated as the distance from the paddle starting position (on the right side of the screen; see Figure 1) to the ball arrival position on the interception axis. Angle of approach (AoA) was operationalized as the angle (in degrees; ranging from −51.2° to +51.2°) under which the ball approached the horizontal interception axis (i.e., the trajectory incidence angle, see Figure 2). Ball flight time (BFT) was considered as a factor (i.e. a nominal variable) with two levels and Group (G) was a factor with three levels. Lastly, participant (P) was considered as random effect.

For the analysis of the presence of observers' “no”-calls, the GLMER model used the logit link function as the distribution was binomial (“no” present vs. absent). To assess the influence of D, BFT, AoA, and G on the judgments provided, we started off with an intercept-only model (i.e., a model with only P as random effect). Using a stepwise forward approach, we added other variables as predictors to the model and retained only those which led to a decrease of the Akaike Information Criterion (AIC) by 2 or more. We considered the VIF (variance inflation factor) for all variables and interactions to check for multicollinearity. After entering the main effects, we tested the various interactions between the variables in the model with main effects for a significant improvement of the model fit. We performed this procedure until the model could not be significantly improved any further.

For the analysis of the timing of observers' “no”-calls, a LMER model was used. In this analysis, the time elapsed after the start of ball motion until the advent of the actor's “no”-calls (A-t_no_) was considered as a predictor variable. Ball flight time (BFT) was considered as a factor with two levels and Group (G) was a factor with three levels. Due to the unbalanced presence of “no”-calls (and thus their timings) across the continuous variables of distance and angle of approach, D and AoA were not included as predictors in this model. Lastly, participant (P) was considered as random effect. The same abovementioned procedure was performed to arrive at the final model. The entire outputs and specifications of each model are available in the Supplementary material.

## 3 Results

### 3.1 Frequency of “no”-calls

Table 1 presents the overall frequency of “no”-calls in the *judging* session per observer and per group. We excluded three trials in which the “no” was called before the ball started moving (i.e., in the absence of any visual information about ball and paddle movement). Henceforth, the analyses of this *judging* session therefore pertain to the remaining trials (*n* = 5,397). Overall, participants made 1,488 “no”-calls out of this total of 5,397 trials, which amounts to a “no”-call in 27.6% of all trials.

**Table 1:** Frequency of “no”-calls per observer per group in the *judging* task.

**Group T + Jf**	**Freq. “no”-calls**	**Group Jf**	**Freq. “no”-calls**	**Group T+Jo**	**Freq. “no”-calls**
P01	14	P13	50	P25	21
P02	60	P14	57	P26	54
P03	61	P15	39	P27	47
P04	33	P16	13	P28	33
P05	57	P17	47	P29	35
P06	41	P18	46	P30	18
P07	36	P19	53	P31	58
P08	64	P20	50	P32	35
P09	39	P21	72	P33	35
P10	47	P22	46	P34	40
P11	22	P23	35	P35	21
P12	43	P24	39	P36	27
Count	517		547		424
Total trials	1,798		1,800		1,799
Percentage	28.75 %		30.39 %		23.57 %

**Figure 3:**
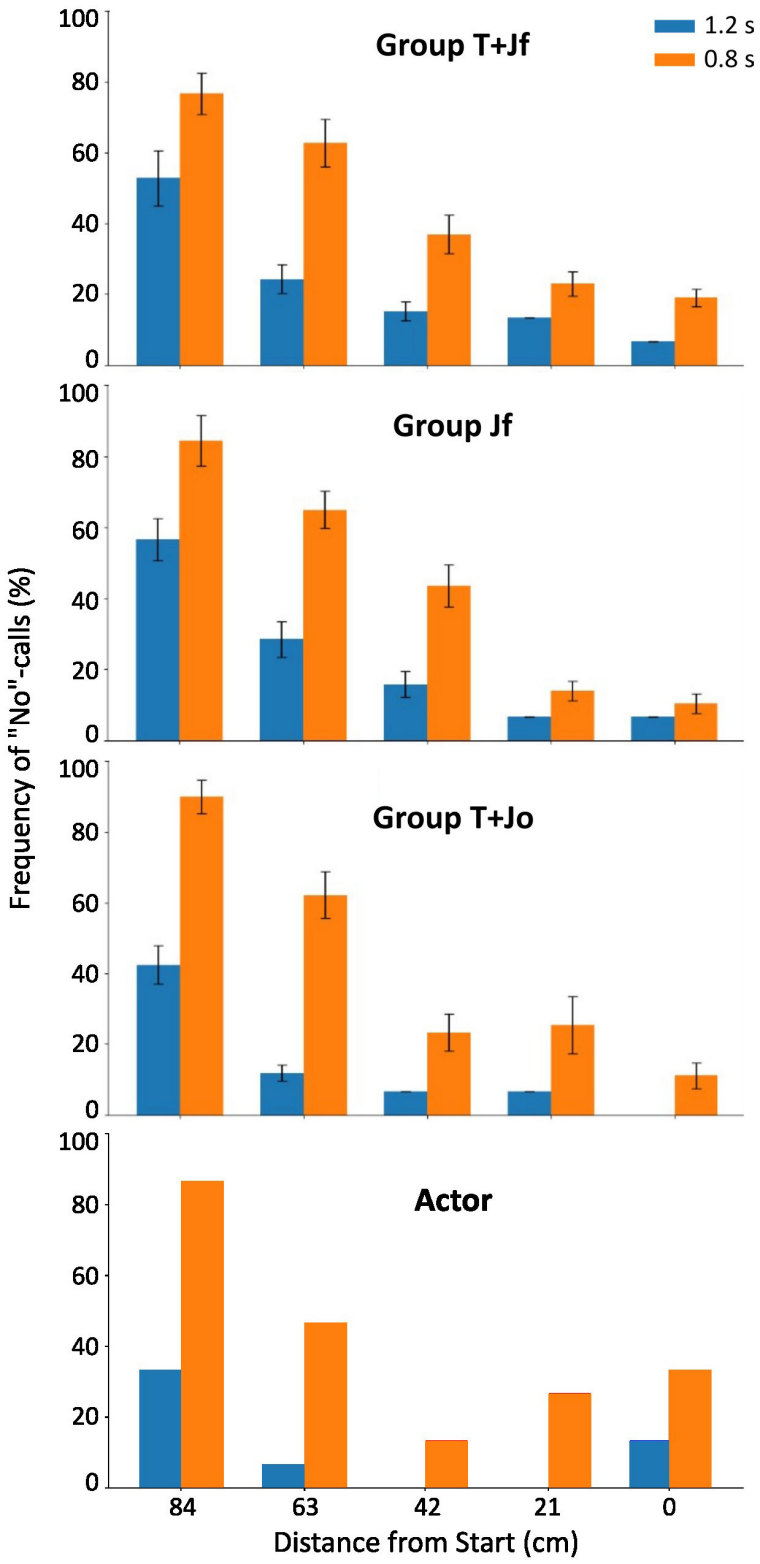
Percentage frequency of “no”-calls as a function of distance from start and ball flight time. The top three panels correspond to the three groups of observers: Group T+Jf, Group Jf and Group T+Jo, respectively. The bottom panel is that of the actor. The two ball flight times are 1.2 s (blue) and 0.8 s (orange). The error bars represent standard error of the means per condition.

As detailed in the Methods section, balls could move down toward different positions on the interception axis, under different angles, with one of two ball flight times. The top three panels of Figure 3 present the average frequencies of the “no”-calls (i.e., verbal judgments) for each group, as a function of (binned) ball arrival distance from the starting position (D) and ball flight time (BFT). The frequency of “no”-calls clearly increased with an increase in distance from the starting position and a decrease in ball flight time. To formally test which task variables played a role in the distribution of “no”-calls from the observers, we performed a Generalized Linear Mixed Effects Regression (GLMER) analysis, in which we considered the variables distance from the starting position (D), ball flight time (BFT), angle of approach of the ball (AoA) as well as group (G) as fixed effects, and participant (P) as random effect (cf. Damle et al., 2024). With all VIF below 2, multicollinearity turned out not to be an issue. The set of significant predictors (i.e., a summary of the final model) is presented in the first column of Table 2 (Observers' Verbal Judgment; for the full model output, see Supplementary Table 1). Apart from the significant effects of the distance from the starting position (D) and ball flight time (BFT), the angle of approach (AoA) also showed up as a significant predictor in the model of the “no”-calls, both as a main effect and in interaction with the distance from the start.

**Table 2:** Factor-wise estimates and standard errors per model for the observers and actor respectively.

**Factor-wise estimates (b) and standard errors of each GLMER model**		
**GLMER model**	**Observers' verbal judgment (*****n*** = **5,397)**	**Actor's verbal judgment (*****n*** = **150)**
Intercept	−1.986 ± 0.1593	X
D	1.778 ± 0.0675	0.809 ± 0.229
BFT (1.2−0.8 s)	−2.223 ± 0.0967	−1.979 ± 0.473
AoA	0.107 ± 0.0578	X
D x AoA	0.305 ± 0.0553	X
G1 (Jf – T+Jf)	X	
G2 (T+Jo – T+Jf)	X	
Equation	Obs. Judg. ~ D + BFT + AoA + D x AoA + (1 | P)	Actor Judg. ~ D + BFT

The first column shows the GLMER results for the observers' verbal judgments. The second column shows the multiple logistic regression result for the actor's verbal judgments. The ball flight time is considered as a factor (BFT) with 0.8 s as the reference level. Group is considered as a factor with two contrasts (G1 and G2), with Group T+Jf as the reference level. For the task variables (D, BFT and AoA) only significant main effects and interactions occurring in at least one of the two models are reported (with an X indicating its absence in one), so as to avoid unnecessary inflation of Table size. The absence of (main) effects of group differences is indicated (by X) for clarity purposes. The observers' mixed effects model includes participant as random intercept, denoted in the equation by (1 | P).

**Figure 4:**
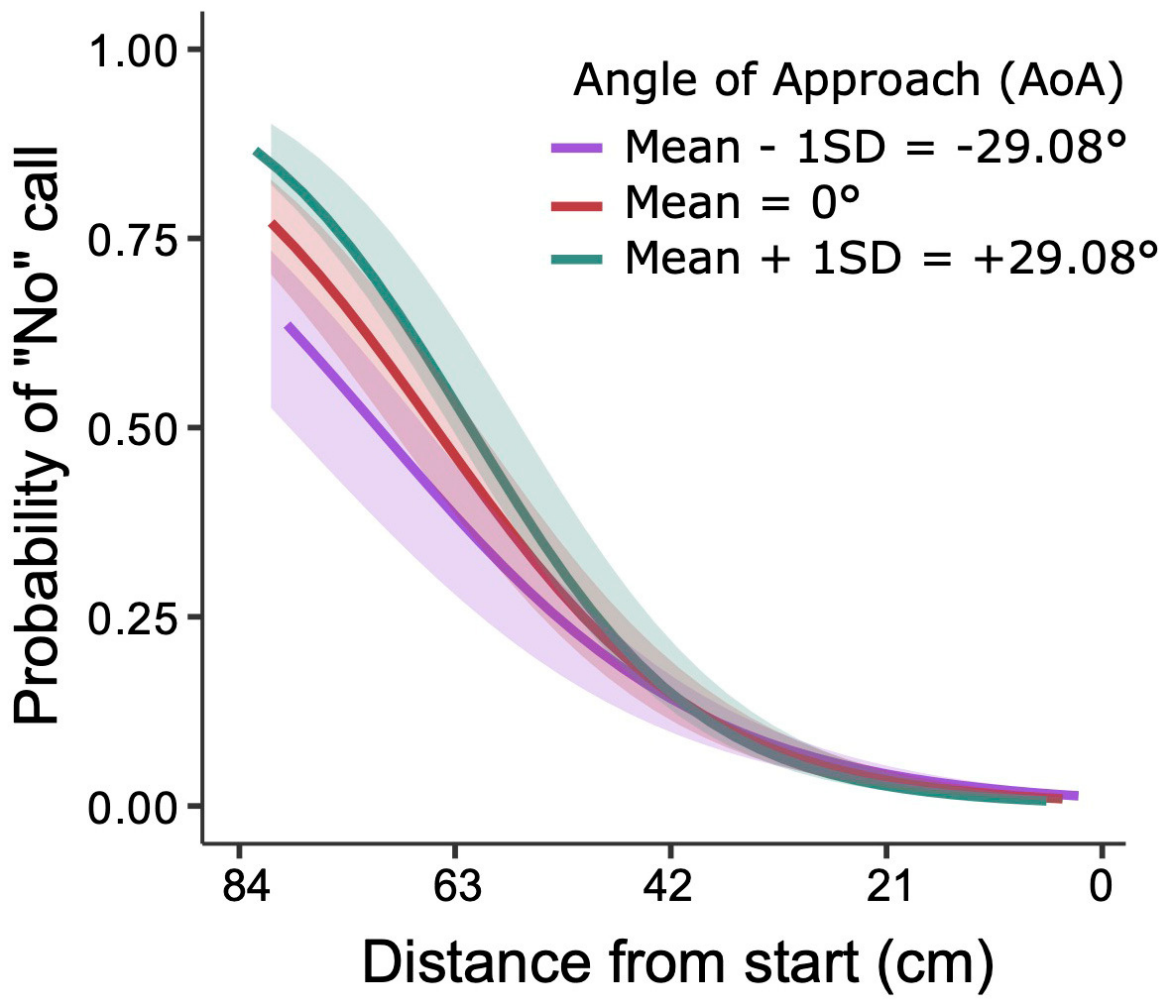
Significant interaction effect of distance from start (D) and angle of approach (AoA) on the probability of “no”-call being made. The X-axis represents the distance to be covered from start. The starting position was on the right side of the X-axis (0 cm). On the Y-axis, a 0 represents an absence of the “no”-call, and 1 represents the presence of a “no”-call. The three curves represent the angles of approach, respectively: negative (purple), zero (red) and positive (cyan). The shaded region around the curves represents the 95% confidence interval.

In line with the observed frequency distributions of “no”-calls (cf. Figure 3), this analysis thus confirms that the probability of “no”-calls increased with increasing distance between the paddle's initial position and the ball arrival position on the interception axis (effect of D) and with decreasing ball flight time (effect of BFT). Furthermore, the model-generated sigmoid probability curves were also affected by the angle of approach of the ball (see Figure 4 for a graphical representation of the D x AoA interaction effect). For positive angles of approach–that is, ball trajectories with a leftward (outward) horizontal velocity component– the curve was shifted slightly to the left, and *vice versa* for negative angles of approach (note that with the distances D in the present study the predicted probability never reached 100%). Finally, we observed no significant effect of group (G) on the presence of a “no”-call, which is why this variable is absent in the final model.

To be able to compare the observers' results with those of the actor, we analyzed the presence of “no”-calls made by the actor herself for the same set of trials. We note that we could not simply adopt the results from Damle et al. (2024), because in the present study we excluded the trials with the fastest ball flight time (BFT = 0.6 s) from the full set of trials from that study (see Methods section). For the present set of trials, the actor called a “no” in 39 out of 150 trials (i.e., 26.0% of the trials). The bottom panel of Figure 3 (“Actor”) presents the frequency of the “no”-calls by the actor, also as a function of (binned) distance from the starting position (D) and ball flight time (BFT). Overall, the patterns of “no”-calls by the actor resembled those provided by the observers (Figure 3, top three panels), except perhaps for the slightly higher number of “no”-calls to the right of the interception axis, near the starting position. Next, we performed a multiple logistic regression analysis of the actor's “no”-calls. Table 2 (second column, Actor's Verbal Judgment; for the full model output, see Supplementary Table 2) shows that, similarly as for the observers, the probability of “no”-calls by the actor increased with increasing distance from the starting position and with shorter ball flight times. The angle of approach did not show up as significant predictor in the final model of the actor, neither as a main effect nor in interaction with distance from the starting position. Probably related to the difference in the number of data points (5,397 for the observers and 150 for the actor), the standard errors of the estimates were considerably larger in the model fit of the actor's data than in the model fit of the observers' data.

### 3.2 Timing of “no”-calls

**Figure 5:**
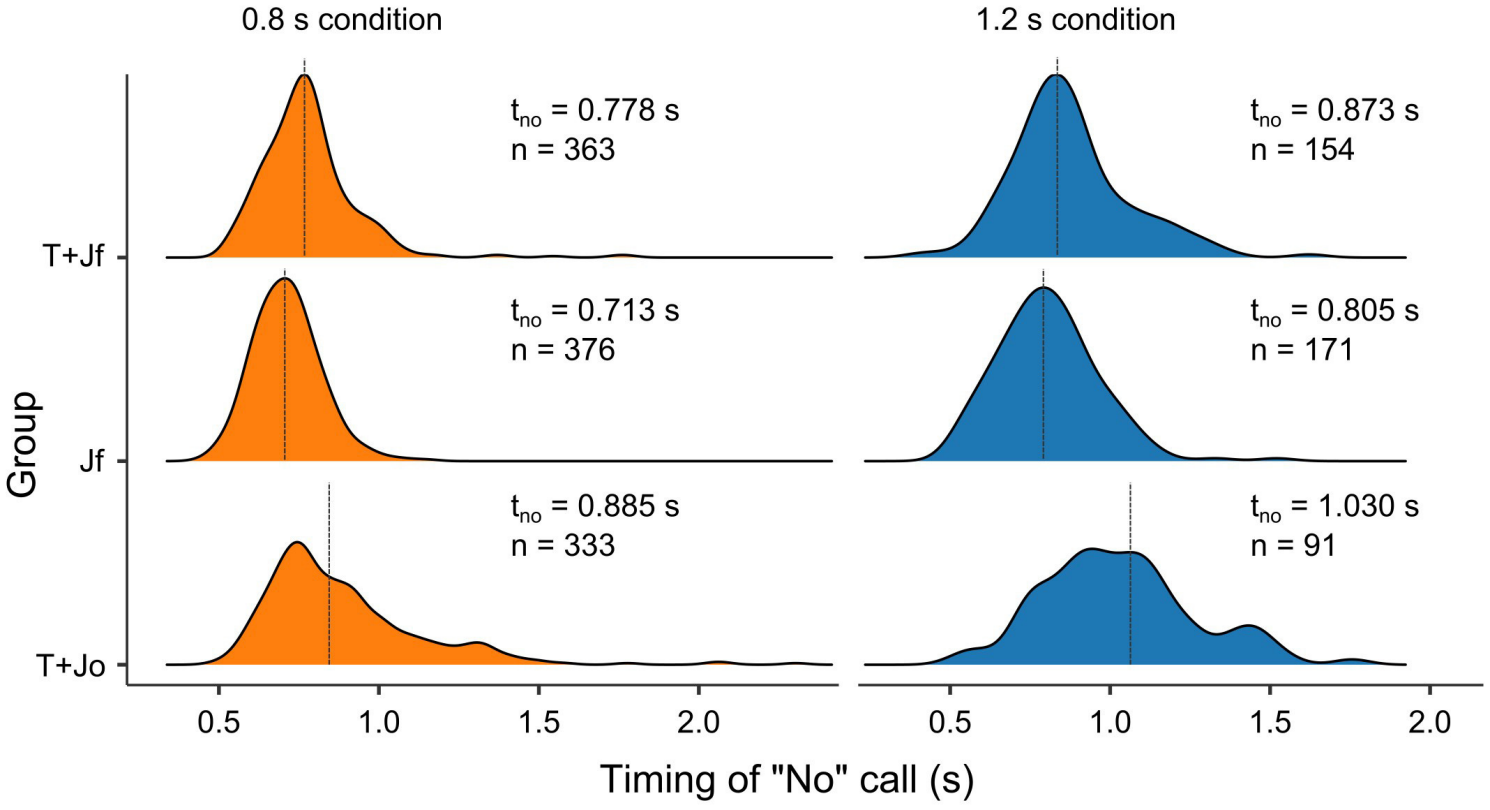
Probability density plots of the observers' timing of calling “no” in seconds, as a function of group and ball flight time. The three groups are Group T+Jf, Group Jf and Group T+Jo respectively. The two ball flight times are 0.8 s (orange) and 1.2 s (blue). Indicated are average times of calling “no” (t_no_) and number of trials (n) for each group and ball flight time.

After considering the variables that were related to whether a “no” was called, we proceeded with identifying the variables that affected the timing of the “no”-calls. Figure 5 presents the distributions and means of the times of calling “no” (t_no_) for the three groups and the two ball flight times. When considering the complete set of “no”-calls, t_no_ was 0.821 ± 0.197 s (*Mean* ±*SD*). Split by group, t_no_ was 0.806 ± 0.167 s for Group T+Jf, t_no_ was 0.742 ± 0.131 s for Group Jf, and t_no_ was 0.916 ± 0.252 s for Group T+Jo, respectively. Observers took longer to make verbal judgments for the longer ball flight time (for BFT = 1.2 s, *t*_no_ = 0.879 ± 0.204 s) relative to the shorter ball flight time conditions (for BFT = 0.8 s, t_no_ = 0.788 ± 0.188 s). When we consider the actor, her “no”-calls, on average, came after 0.773 ± 0.189 s (t_no_ = 0.857 ± 0.210 s for BFT = 1.2 s and t_no_ = 0.752 ± 0.177 s for BFT = 0.8 s, respectively).

We tested which variables played a role in the timing of “no”-calls from the observers via a Linear Mixed Effects Regression (LMER) analysis, in which we considered the timing of the actor's “no”-calls (A-t_no_), ball flight time (BFT) and group (G) as fixed effects, and participant (P) as random intercept. Again, collinearity did not turn out to be an issue. As can be seen from Table 3 (for the full model output, see Supplementary Table 3), the factors A-t_no_, BFT and G emerged all three as significant predictors. Comparisons of the timing of the “no”-calls across the three groups showed that Group T+Jo differed significantly from Group T+Jf. Occluding the final part of the animations led to significantly later “no”-calls. However, the analysis did not indicate a significant difference between Group T+Jf and Group Jf, with these groups differing in having or not having had training on the lateral interception task before the *judging* session, respectively.

**Table 3:** LMER model of the timing of the observers' verbal judgments (“no”-calls).

**Factor-wise estimates (b) and standard errors**	
Intercept	0.8412 ± 0.0178
A-t_no_	0.0692 ± 0.0287
BFT (1.2 - 0.8 s)	0.1351 ± 0.0142
G1 (Jf – T+Jf)	X
G2 (T+Jo – T+Jf)	0.0886 ± 0.0422
Equation	Timing Obs. ~ A-t_no_ + BFT + G2 + (1 | P)

The ball flight time is considered as a factor (BFT) with 0.8 s as the reference level. Group is considered as a factor with two contrasts (G1 and G2), with Group T+Jf as the reference level. The timing of actor's “no”-calls (A-t_no_) is included as a continuous variable. Only significant main effects and interactions contributing to the models are reported, with their absence indicating that no significant interactions were found. The observers' mixed effects model includes participant as random intercept, denoted in the equation by (1 | P).

## 4 Discussion

In the present study, we set out to investigate whether observers can perceive the affordance of interceptability for another person in a manual lateral interception task. In a previous study in which we adopted this task, we demonstrated how people are able to perceive interceptability for themselves (Damle et al., 2024). Here, we presented recorded ball and paddle kinematics of interceptive actions of one of the participants of this previous study (the actor) to a new group of participants (the observers). Just as we had asked the participants in the previous study to indicate, during interception attempts, that they felt that they would not be able to intercept a ball (by calling “no”), here we asked the analogous question but now concerning the interceptability for the actor. We thus asked the observers to indicate, on each trial, whether the actor could intercept the ball and recorded the observers' verbal judgments (“no”-calls).

The frequencies of the “no”-calls, the pattern of the “no”-calls and the timing of the “no”-calls of the observers, overall, resembled those of the actor. Observers indicated non-interceptability in approximately 28% of the trials, as compared to the 26% of the same set of trials by the actor herself. When we consider the pattern of “no”-calls, for both the actor and the observers, the frequency of “no”-calls increased with increasing distance from the paddle starting position and decreasing ball flight time (i.e., increasing vertical ball speed). As for the timing of the “no”-calls, both the actor and the observers took 0.7–0.8 seconds, on average, to indicate that a ball was uninterceptable.

While the (task variable-dependent) patterns of the observers' results generally resembled those of the actor, there were also some nuanced distinctions. When comparing the significant predictors of the probability of “no”-calls by the observers and actor, differences showed up in the presence of an effect of the balls' angle of approach, as a main effect and in interaction with the balls' arrival distance from the starting position. Whereas for the observers, the “no”-calls were slightly affected by the angle under which balls approached the interception axis, the analyses did not reveal the same effect for the actor (see Table 2). One obvious reason could be the difference in number of data points used in the analyses of the observers' “no”-calls (based on 5397 trials) and the actor's “no”-calls (based on 150 trials). The actor was selected from a set of 15 participants in the Damle et al. (2024) study. The similar analysis (with all 15 participants but also with the additional trials with shorter ball flight times) did show a similar interaction effect of angle of approach and distance from the starting position as we found for the observers in the present study, although the sign of the main effect of the angle of approach in the Damle et al. (2024) modeled results was different from the sign of the main effect of the observers in the present study's modeled “no”-calls. For the observers, the frequency of “no”-calls tipped slightly toward positive angles of approach whereas for the actors in the Damle et al. (2024) study, the frequency of “no”-calls was slightly larger for negative angles of approach. A clear explanation of this slight difference probably needs future study.

We have taken our findings that the judgments for another person as made by the observers in our study were similar to those of previous participants who made analogous judgments, for themselves, while performing the interception attempts, to imply that these observers were able to perceive the affordance of interceptability for another (in this study, for the actor that we had selected). Their “no”-calls, in the majority of cases, came before the final result had become clear. Rather than reporting retrospectively on a seen event, they prospectively perceived the vanished opportunity for successful completion of the action. What we presented to the observers were motions of a rectangle (paddle) and a circle (ball) on a large screen. Importantly, the relative motion of these elements was structured just as it would when the rectangle would be controlled by an agent (e.g., Damle et al., 2024; Ledouit et al., 2013, 2024). We contend that it is in this relational structure that the information should reside that specifies this to be a failed or successful interception attempt (cf. Gibson, 1979/1986; Baggs and Raja, 2024). The relational structure in point-light displays representing biological motion has been implied to be informative of affordances for others before (Weast et al., 2014; Weast-Knapp et al., 2019). Here, we suggest that the information for interceptability is to be found in the time evolution of the angle β formed by the horizontal and the (invisible) line connecting the ball and the paddle. In previous studies on *doubles pong* (Benerink et al., 2016, 2018; Van Opstal et al., 2018), two players, in a similar setup as ours, could both move a paddle horizontally along a shared interception axis. Their task was to make sure that one of the two realized the interception without the two paddles colliding. It turned out that the coordination between the two players could well be understood by one player leaving the interception to the other, even when both had initiated an interception attempt. A positive rate of change of angle β informed the yielding player that the other player was underway to arrive at the interception location on time. Future studies are needed to show how (change over time in) angle β might also specify interceptability.

As mentioned before, our inferences regarding people's ability to perceive the affordance for others was based on the similarity of the task variable patterns affecting the “no”-calls (in terms of both their presence and timing) when participants indicated uninterceptabilityfor themselves (Damle et al., 2024) or for another person (the present study). One might wonder why we did not also consider the accuracy of the “no”-calls here. In fact, assessing accuracy in dynamic settings is not straightforward, in the sense that a given combination of task variables does not fully define whether a ball is interceptable or not for a given person. Indeed, as already hinted at in the introduction, in dynamic settings the same person could be able to intercept a ball that will arrive at a given position on the interception axis after a given ball flight time on one occasion and not be able to intercept this same ball on another occasion, due to variations in how the person behaved early on in a trial. A ball arriving after a short flight duration at a position relatively far from the paddle starting position may be interceptable if the person initiates a high-velocity movement early on. Initiating the movement a little later may no longer allow interception; indeed, the affordance of interceptability may then have vanished. Task variables thus only roughly define (un) interceptability. For lack of an alternative, in the previous Damle et al. (2024) study we used a (task variables-based) GLMER model of the successes and failures of participants interception attempts in one (*action*) session to assess the accuracy of “no”-calls in a next (*judging*) session. Applying this same method here, now necessarily with much fewer data points (trials) available from the unique actor selected to build the model, was deemed to be a too poor basis to evaluate the accuracy of observers' “no”-calls.

In the present study, we also examined the effect of two manipulations on the perception of interceptability for another, namely prior training (practice) and late occlusion of the visual screen. Although these manipulations were largely exploratory in nature, we expected that prior training and late occlusion would improve and impair the observers' affordance perception for the actor, respectively. In contrast, we found no significant effects of providing training on the observers' affordance judgments for the actor and an effect of late occlusion only on the timing of the judgments. We discuss each effect in further detail below.

Previous research has shown that prior training improves affordance perception. However, this has been studied majorly regarding one's own affordances. This positive effect of training (or practice) has been established in the perception of passability through doors (Franchak et al., 2010; Yasuda et al., 2014), minimum height of passage (Stoffregen et al., 2009), throwing and walking abilities (Hospodar et al., 2023), and climbers' perception of grasp-ability (Seifert et al., 2018; but see Walsh et al., 2024). However, the present study considered affordances for another, for which, as far as we are aware of, the effect of specific training for the observer has not been studied. A few studies did look at the effect of expertise on the affordance perception for another. For instance, Weast et al. (2011, 2014) studied the role of athletic experience in affordance perception of another actor's maximum vertical jumping height and horizontal long-jumping distance. They found that one's own sports experience enhances perceptual judgments for others for affordances that are relevant for the specific sports. If expertise would also improve perception of affordances for other individuals in lateral interception, our results might indicate that the expertise was already sufficiently present or that the training was not extensive enough to improve the expertise sufficiently.

Secondly, we occluded the final part of the ball and paddle motion for one group of the observers. We expected some worsening of affordance judgments of the observers, but we did not see significant differences between the groups with and without late occlusion of the visual screen. However, the timing of the “no”-calls was significantly slower with late occlusion. Although one might have expected that occlusion would have led the observers to give their verbal judgments right after the occlusion happened (e.g., see Fajen et al., 2011), this was not what the observers in the present study seem to have been doing. Considering the distributions of the timing of the verbal judgments (Figure 5), rather, they seemed to have been delaying their responses in comparison to the observers without the occlusion. In line with instructions, the latter probably felt more compelled to call their “no”-s before the ball reached the interception axis. Still, the observers showed similar performance with and without occlusion, suggesting that the information for uninterceptability was available well before the moment of occlusion, which was well before balls reached the interception axis.

In conclusion, while Damle et al. (2024) showed that individuals are well able to indicate uninterceptability for themselves in our lateral-interception task, the present study provided the first evidence that people can also see whether balls are interceptable or not for another person. In the present study, we used the ball and paddle kinematics from one selected actor from our previous study. With this basis for the displays presented to our groups of observers, theoretically, it could be the case that the observers learned to know the action capabilities of this single person rather than showing that the kinematics contain information of interceptability for others in general. With this in mind, it seems fair to claim that the present study is a useful first step at demonstrating the ability of perceiving interceptability for another.

## Data Availability

The raw data supporting the conclusions of this article will be made available by the authors, without undue reservation.
