# Supplementary material for: Perceiving the affordance of interceptability for another
Source: Front Psychol. 2025 Jun 3;16:1566278. doi: 10.3389/fpsyg.2025.1566278 (PMC12171503; doi:10.3389/fpsyg.2025.1566278)
Supplement: Supplementary file 1 [file Table_1.pdf]

Supplementary Material

Table S1: GLMER analysis on observers' verbal judgment. Abbreviations: D: distance from start, BFT: ball flight time, and AoA: angle of approach.

Model Info

Info	Value	Comment
Model Type	Logistic	Model for binary y
Call	glm	Obs. Judg. ~ D + BFT + AoA + D x AoA + (1 | P)
Link function	Logit	Log of the odd of y=1 over y=0
Direction	P(y=1)/P(y=0)	P(Verbal Judg. = present) / P(Verbal Judg. = absent)
Distribution	Binomial	Dichotomous event distribution of y
LogLikel.	-1912.220	Unconditional Log-Likelihood
-2*LogLikel.	3824.440	Unconditional absolute deviance
Deviance	3698.150	Conditional relative deviance
R-squared	0.537	Marginal
R-squared	0.625	Conditional
AIC	3836.440	Less is better
BIC	3876.002	Less is better
Residual DF	5391.000	
Chi-squared/DF	0.943	Overdispersion indicator
Converged	yes	
Optimizer	bobyqa	

Model Results

Fixed Effect Omnibus tests

	X^2	df	p
D	693.90	1.00	< .001
BFT	528.19	1.00	< .001
AoA	3.44	1.00	0.064
D ✻ AoA	30.35	1.00	< .001

Fixed Effects Parameter Estimates

				95% Confidence Interval				
**Names**	**Effect**	**Estimate**	**SE**	**Lower**	**Upper**	**exp(B)**	**z**	**p**
(Intercept)	(Intercept)	-1.986	0.1593	-2.29829	-1.674	0.137	-12.46	< .001
D	D	1.778	0.0675	1.64562	1.910	5.917	26.34	< .001
BFT	1.2 - 0.8	-2.223	0.0967	-2.41284	-2.034	0.108	-22.98	< .001
AoA	AoA	0.107	0.0578	-0.00613	0.220	1.113	1.85	0.064
D ✻ AoA	D ✻ AoA	0.305	0.0553	0.19630	0.413	1.356	5.51	< .001

Random Components

Groups	Name	SD	Variance	ICC
Participant	(Intercept)	0.875	0.766	0.189
Residuals		1.000	1.000	.

Note. Number of Obs: 5397, groups: Participant 36

Post Hoc Tests

Post Hoc Comparisons - BFT

Comparison						
**BFT**		**BFT**	**exp(B)**	**SE**	**z**	**$p_{bonferroni}$**
0.8	-	1.2	9.24	0.894	23.0	< .001

Table S2: Multiple logistic regression on actor's verbal judgment. Abbreviations: D: distance from start, BFT: ball flight time.

Model Fit Measures

Model	Deviance	AIC	R^2_{McF}
1	138	144	0.196

Model Coefficients

Predictor	Estimate	SE	Z	p
Intercept	-0.403	0.253	-1.59	0.111
D	0.809	0.229	3.53	< .001
BFT:				
1.2 – 0.8	-1.979	0.473	-4.19	< .001

Note. Estimates represent the log odds of "Verbal Judg. present = 1" vs. "Verbal Judg. absent = 0"

Table S3: LMER analysis on observers' timings of "no"-calls. Abbreviations: A-t_{no}: Actor's timings of "no"-calls, BFT: ball flight time, G: Group.

Model Info

Info	
Estimate	Linear mixed model fit by REML
Call	Timing Obs. ~ A-t_{no} + BFT + G2 + (1 | P)
AIC	-671.773
BIC	-611.474
LogLikel.	329.058
R-squared Marginal	0.172
R-squared Conditional	0.422
Converged	yes
Optimizer	bobyqa

Fixed Effect Omnibus tests

	**F**	**Num df**	**Den df**	**p**
A-t_{no}	5.83	1	746.8	0.016
G	6.20	2	32.7	0.005
BFT	89.90	1	755.7	< .001

Note. Satterthwaite method for degrees of freedom

Fixed Effects Parameter Estimates

				95% Confidence Interval				
**Names**	**Effect**	**Estimate**	**SE**	**Lower**	**Upper**	**df**	**t**	**p**
(Intercept)	(Intercept)	0.8412	0.0178	0.80630	0.8760	37.1	47.31	< .001
A-t_{no}	A-t_{no}	0.0692	0.0287	0.01304	0.1254	746.8	2.41	0.016
BFT	1.2 – 0.8	0.1351	0.0142	0.10714	0.1630	755.7	9.48	< .001
G1	Jf – T+Jf	-0.0593	0.0423	-0.14222	0.0236	32.8	-1.40	0.170
G2	T+Jo – T+Jf	0.0886	0.0422	0.00597	0.1712	32.7	2.10	0.043

Random Components

**Groups**	**Name**	**SD**	**Variance**	**ICC**
Participant	(Intercept)	0.0976	0.00953	0.301
Residual		0.1486	0.02209	

Note. Number of Obs: 783, groups: Participant 36

Post Hoc Tests

Post Hoc Comparisons - BFT

**Comparison**							
**BFT**		**BFT**	**Difference**	**SE**	**t**	**df**	**$p_{bonferroni}$**
0.8	-	1.2	-0.135	0.0142	-9.48	756	< .001
